# Comparison of the predictive value of scoring systems on the prognosis of cirrhotic patients with suspected infection

**DOI:** 10.1097/MD.0000000000011421

**Published:** 2018-07-13

**Authors:** Peng Lan, Shuo-Jia Wang, Qiu-Cheng Shi, Ying Fu, Qing-Ye Xu, Tao Chen, Yun-Xian Yu, Kong-Han Pan, Ling Lin, Jian-Cang Zhou, Yun-Song Yu

**Affiliations:** a Department of Critical Care Medicine, Sir Run Run Shaw Hospital; b Department of Epidemiology and Health Statistics, School of Public Health; c Department of Infectious Disease; d Department of Clinical Laboratory, Sir Run Run Shaw Hospital, Zhejiang University School of Medicine, Hangzhou, Zhejiang, China.

**Keywords:** cirrhosis, infection, prognosis, scoring system

## Abstract

Cirrhotic patients with infection are prone to develop sepsis or even septic shock rendering poorer prognosis. However, few methods are available to predict the prognosis of cirrhotic patients with infection although there are some scoring systems can be used to predict general patients with cirrhosis. Therefore, we aimed to explore the predictive value of scoring systems in determining the outcome of critically ill cirrhotic patients with suspected infection.

This was a retrospective cohort study based on a single-center database. The prognostic accuracy of the systemic inflammatory response syndrome (SIRS) criteria, quick Sequential Organ Failure Assessment (qSOFA), chronic liver failure (CLIF)-SOFA, quick CLIF-SOFA (qCLIF-SOFA), CLIF-consortium organ failure (CLIF-C OF), Model for End-Stage Liver Disease (MELD), and Simplified Acute Physiology Score (SAPS) II were compared by using area under the receiver operating characteristic (AUROC) curve and net benefit with decision curve analysis. The primary endpoint was in-hospital mortality while the secondary endpoints were duration of hospital and intensive care unit (ICU) stay and ICU mortality.

A total of 1438 cirrhotic patients with suspected infection were included in the study. Nearly half the patients (50.2%) were admitted to the ICU due to hepatic encephalopathy and the overall in-hospital mortality was 32.0%. Hospital and ICU mortality increased as the score of each scoring system increased (*P* < .05 for all trends). The AUROC of CLIF-SOFA (AUROC, 0.742; 95% confidence interval, CI, 0.714–0.770), CLIF-C OF (AUROC, 0.741; 95% CI, 0.713–0.769), and SAPS II (AUROC, 0.759; 95% CI, 0.733–0.786) were significantly higher than SIRS criteria (AUROC, 0.618; 95% CI, 0.590–0.647), qSOFA (AUROC, 0.612; 95% CI, 0.584–0.640), MELD (AUROC, 0.632; 95% CI, 0.601–0.662), or qCLIF-SOFA (AUROC, 0.680; 95% CI, 0.650–0.710) (*P* < .05 for all). In the decision curve analysis, the net benefit of implementing CLIF-SOFA and CLIF-C OF to predict the prognosis of cirrhotic patients with suspected infection were higher compared with SIRS, qSOFA, MELD, or qCLIF-SOFA.

CLIF-SOFA and CLIF-C OF scores, as well as SAPS II were better tools than SIRS, qSOFA, MELD, or qCLIF-SOFA to evaluate the prognosis of critically ill cirrhotic patients with suspected infection.

## 1 Introduction

Liver cirrhosis is a common disease and decompensated cirrhosis is frequently complicated with infection.^[1]^ Surprisingly, infection rates in hospitalized patients with cirrhosis were 4- to 5-fold higher than those among the general patient population, and associated with poor prognosis.^[2]^ Given that cirrhosis is commonly associated with some immune dysfunction, once infected, the cirrhotic patients are prone to develop severe sepsis and septic shock.^[3]^ Despite the advances in clinical care and treatment, the mortality rate of sepsis and septic shock remained 20% to 60%.^[4]^ To more accurately stratify the risk of patients with sepsis, in 2016, Sepsis 3 definition was proposed and Sequential Organ Failure Assessment (SOFA) score was recommended to define sepsis while quick SOFA (qSOFA) was used to identify sepsis patients in ward and emergency department early.^[5]^ Thereafter, systemic inflammatory response syndrome (SIRS), SOFA and qSOFA were validated for sepsis patients in intensive care unit (ICU) and emergency department in different countries.^[6–8]^

On the other hand, for general cirrhotic patients, some liver-specific scores such as Model for End-Stage Liver Disease (MELD),^[9]^ chronic liver failure (CLIF)–SOFA,^[10]^ and CLIF-consortium organ failure (CLIF-C OF)^[11]^ have long been used to evaluate patients’ outcome. Moreover, recently, the quick CLIF-SOFA (qCLIF-SOFA)^[12]^ presented good discriminative ability for outcome of cirrhotic patients. However, for cirrhotic patients with suspected infection, there was no unanimous consent on the superiority of these scores when predicting the prognosis of these patients. Hence, we sought to answer the question by comparing the prognostic accuracy of the abovementioned scoring systems using MIMIC (Medical Information Mart for Intensive Care) III database.

## 2 Materials and methods

### 2.1 Data source and extraction

This study was based on a publicly available ICU database named MIMIC-III (version 1.4), a large, single-center database containing information of more than 40,000 patients admitted to Beth Israel Deaconess Medical Center (a teaching hospital of Harvard Medical School in Boston, MA) from 2001 to 2012. The database contains data of general information, treatment processes, and survival data. The access of the MIMIC III database for research was approved by the Institutional Review Boards of Beth Israel Deaconess Medical Center after completion of the NIH web-based course named “Protecting Human Research Participants.” Since all patients were de-identified, informed consent was waived. Data was extracted from MIMIC-III by using structure query language (SQL) with pgAdmin4 PostgreSQL 9.6.

### 2.2 Inclusion criteria and definitions

Patients with liver cirrhosis and suspected infection were included. Cirrhotic patients were extracted according to International Classification of Diseases (ICD)-9 codes (5712, 5715, 5716 indicated “alcoholic cirrhosis of liver,” “cirrhosis of liver without mention of alcohol,” and “biliary cirrhosis,” respectively). Of the cirrhotic patients, those with suspected infection were extracted if one of the following criteria was fulfilled: ICD-9 contained any of the following term “infection,” “pneumonia,” “meningitis,” “peritonitis,” “bacteremia,” “sepsis,” or “septic”; positive microbiological culture.^[13]^ Of all the cirrhotic patients, those diagnosed with hepatic encephalopathy (HE) was identified by ICD-9 code of 5722. According to previous studies,^[14,15]^ Glasgow Coma Scale (GCS) was associated with West-Haven grade to some degree. Given GCS is somewhat consistent with West Haven grade,^[15]^ thus, in this study, HE patients with GCS 15 and 3 to 5 scores were categorized as grade 1 and 4, respectively, while those with GCS 13 to 14 and 6 to 12 scores fell into grade 2 and 3, respectively.

Demographic, laboratory, and clinical data on ICU admission were collected including age, gender, ethnicity, admission location, Simplified Acute Physiology Score (SAPS) II, comorbidities, complication of cirrhosis, in-hospital and ICU outcomes, some laboratory, and clinical parameters. Prognostic scoring systems including SAPS II, CLIF-SOFA, SIRS, qSOFA, qCLIF-SOFA, MELD, and CLIF-C OF were calculated for all patients (Table 1).^[5,10,11,12,16,17]^ CLIF-SOFA and CLIF-C OF shares the 6 components including bilirubin, kidney, HE grades, international normalized ratio (INR), circulation, and lungs. However, subscores of each component ranged from 0 to 4 for CLIF-SOFA, whereas from 1 to 3 for CLIF-C OF.^[10,11]^ As for qCLIF-SOFA, it included bilirubin, creatinine, INR, mean arterial pressure and vasopressin usage and each with subscores of 0 or 1.^[12]^ Recently proposed qSOFA score contained respiratory rate, mentation status and systolic blood pressure.^[5]^

**Table 1:**
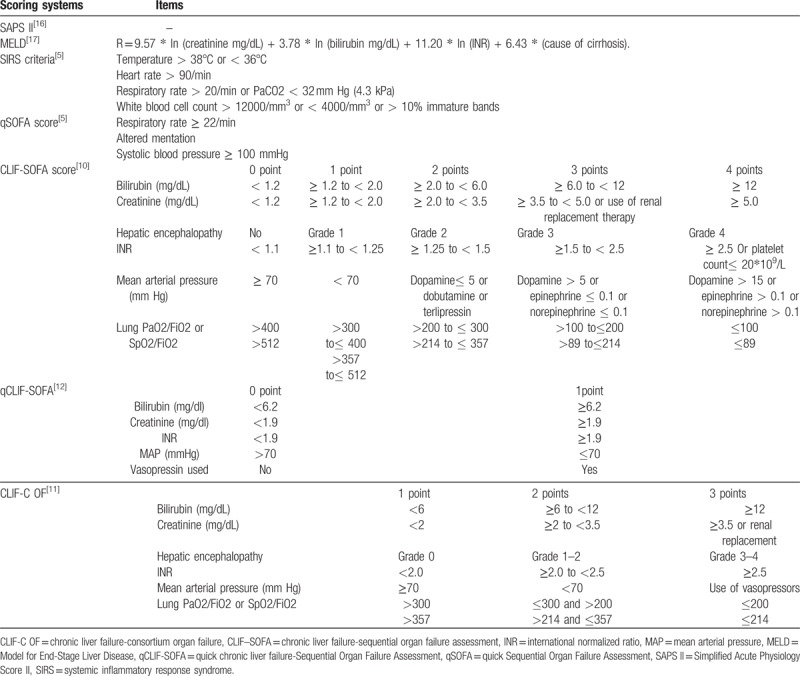
Components of the seven scoring systems.

The primary endpoint was in-hospital mortality. The secondary endpoints included ICU mortality and the length of stay in ICU and hospital.

### 2.3 Statistical analysis

The Kolmogorov–Smirnov test and histograms were performed to test the normality of the distribution of quantitative variables. Normally distributed quantitative variables were presented as mean ± SD while skewed variables were summarized as median and interquartile range (IQR). For comparison, *χ*^*2*^ analysis or Fisher exact test were performed for categorical variables. Quantitative variables were compared using analysis of variance (ANOVA) or *t* test for normally distributed data and Kruskal–Wallis test or Mann–Whitney test for non-normally distributed data.

Predictive accuracy of each score was determined by comparing the area under the receiver operating characteristic (AUROC) curve. Clinical significance and net benefit were estimated by decision curve analysis. Decision curve analysis was first proposed in 2006 and has been used in studies published in the *Lancet*^[18]^ and *Journal of Clinical Oncology*.^[19]^ Decision curve is a complement to ROC curve for a risk model. It is more informative than an ROC curve because the true- and false-positive fractions are displayed as functions of the risk threshold, whereas the risk threshold is suppressed in the ROC curve.^[20]^

All analyses were performed using R 3.3.3 (http://www.r-project.org/), and a *P*-value less than .05 was considered statistically significant.

## 3 Results

### 3.1 Basic characteristics

A total of 1438 ICU patients with cirrhosis and suspected infection were included in the present study (Table 2). Of them, about 64% were male and nearly half aged over 60 years old. In the cohort, the median SAPS II was 56 and the in-hospital mortality was 32.0% and nearly half the patients (50.2%) admitted to ICU for hepatic encephalopathy. Among all the cirrhosis patients, most of them were with sepsis according to Sepsis-3 definition. Approximately 30% patients had associated diabetes mellitus, and 29% patients’ alcohol abuse or dependence. With respect to laboratory data, white blood cell (WBC) count, creatinine, lactate, INR, partial thromboplastin time, total bilirubin, and aspartate aminotransferase were significantly higher for non-survivors than survivors (*P* < .001 for all).

**Table 2:**
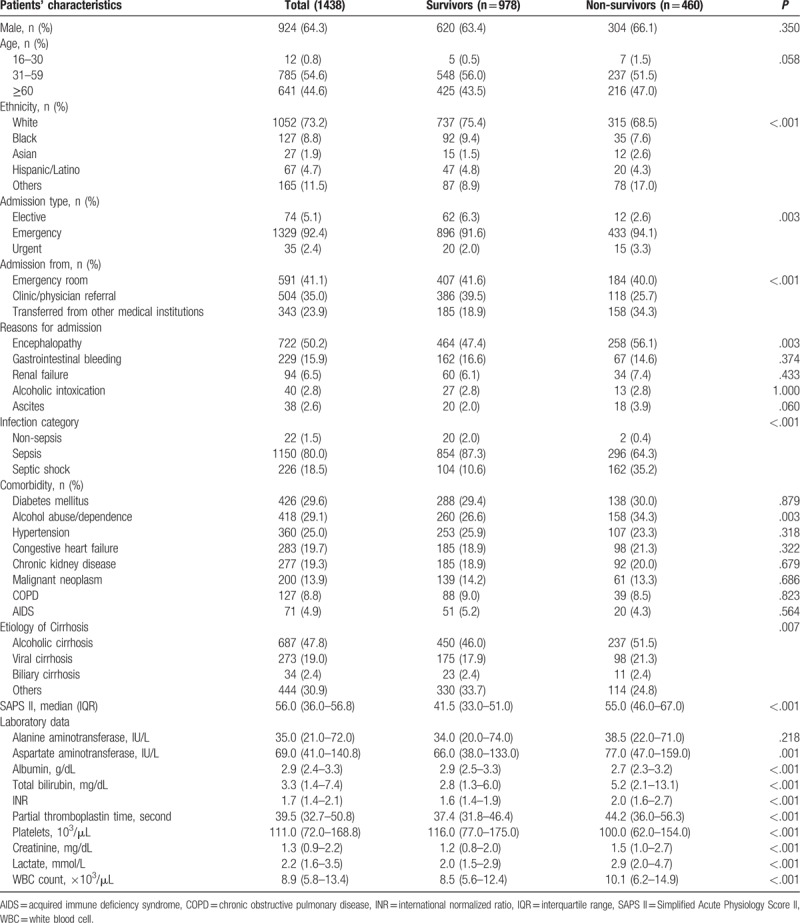
Baseline characteristics of critical ill cirrhotic patients with suspected infection.

The incidence of respiratory, bloodstream, and peritoneal infections was significantly increased among non-survivors (*P* < .05 for all). In this regard, causative agents for worse outcome were predominantly fungal infections (*P* < .001), for example, *Candida albicans* (*P* = .013) and *Aspergillus* (*P* = .001) (Table 3).

**Table 3:**
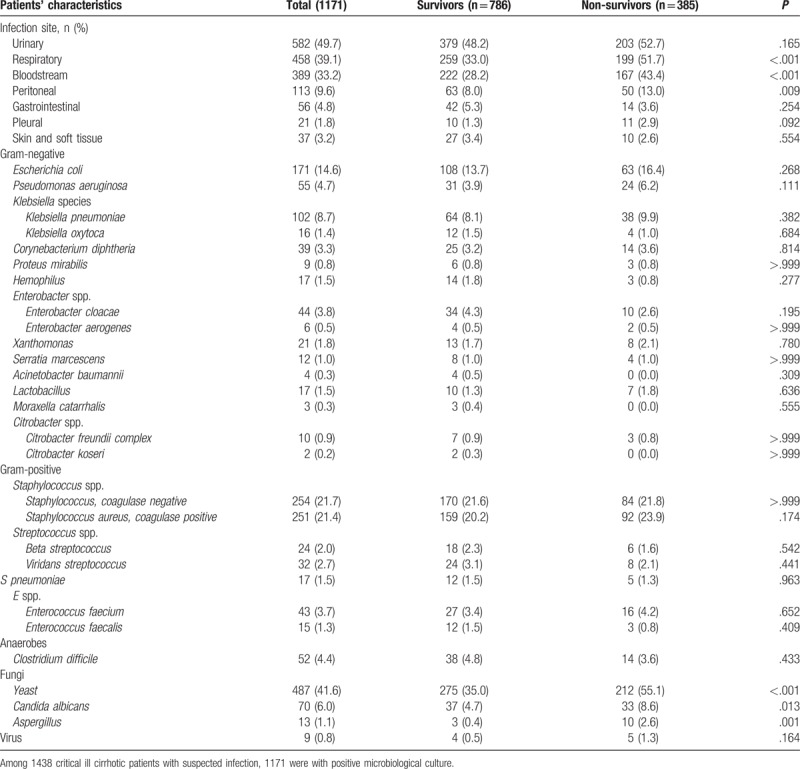
Infection site and causative agents of critical ill cirrhotic patients with suspected infection (n = 1171).

### 3.2 Outcomes of all patients

**Figure 1:**
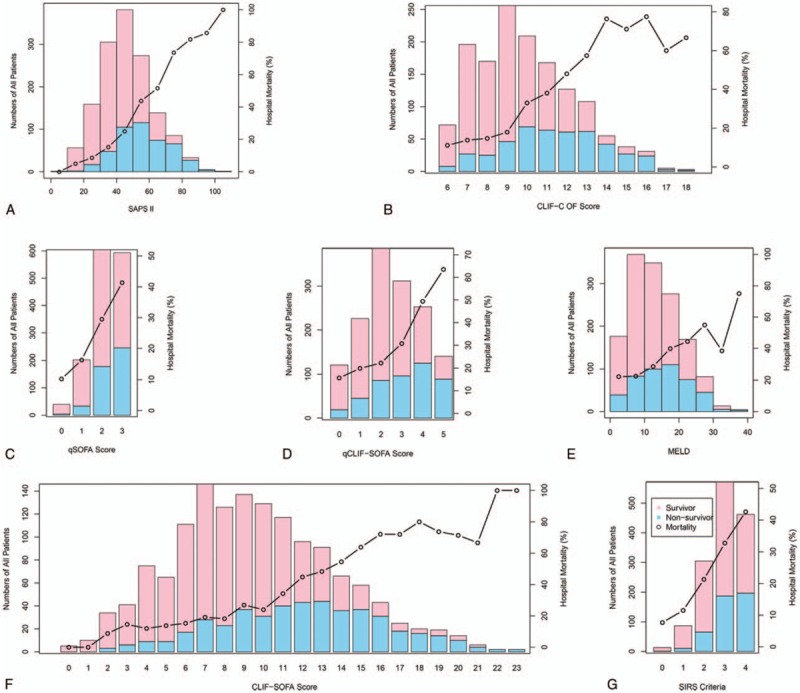
Distribution of all the patients and in-hospital mortality according to the score levels of each scoring system. CLIF-C OF = CLIF-consortium organ failure, CLIF-SOFA = chronic liver failure-SOFA, MELD = Model for End-Stage Liver Disease, qCLIF-SOFA = quick CLIF-SOFA, qSOFA = quick Sequential Organ Failure Assessment, SAPS II = Simplified Acute Physiology Score II, SIRS = systemic inflammatory response syndrome.

The distributions of each scoring system and their association with in-hospital mortality were shown in Figure 1. As expected, in-hospital and ICU mortality elevated as the score of each scoring system increased (*P* < .05 for all trends) (Table 4).

**Table 4:**
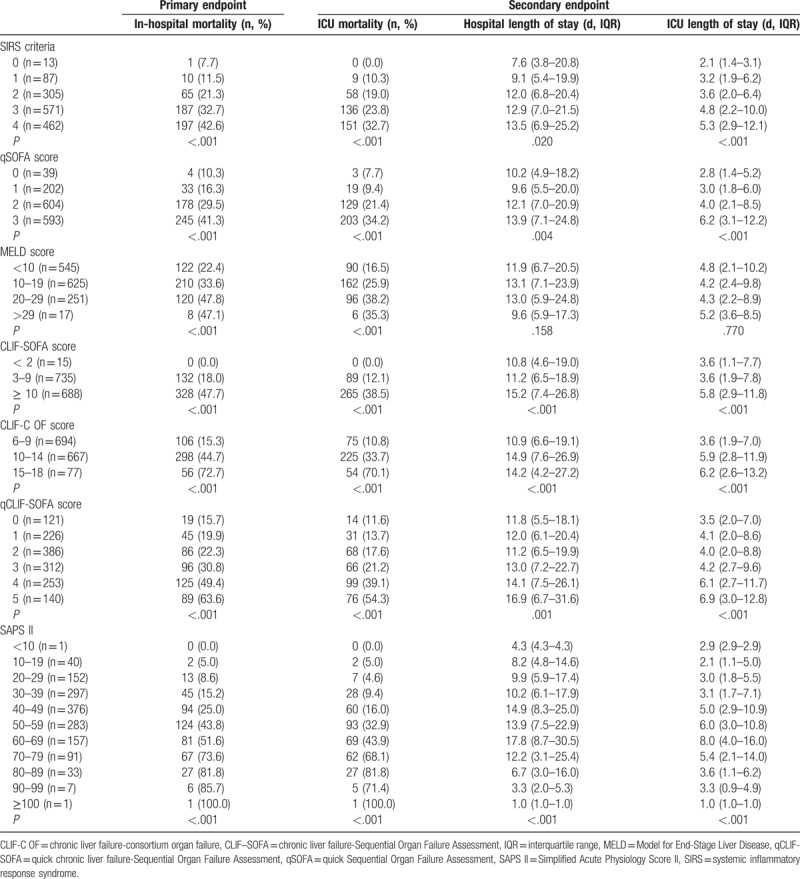
Primary and secondary endpoints based on the score of each scoring system.

### 3.3 Prognostic accuracy of scoring systems

**Figure 2:**
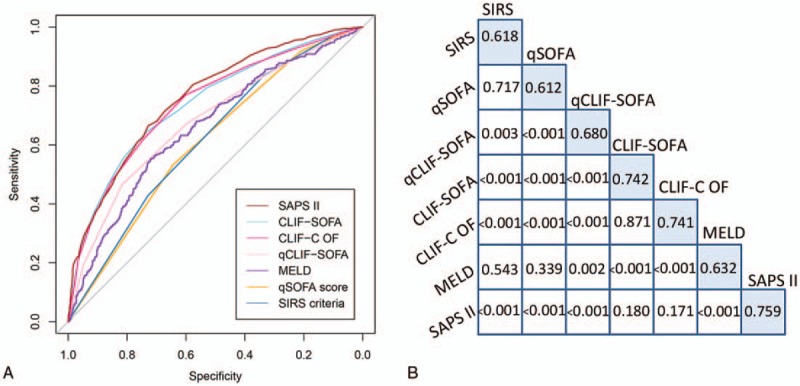
Receiver operating characteristic curve showing the comparison of AUROC of each score (A); the skyblue-shaded diagonal cells indicated the AUROC of each score and below the AUROC data cells are p values for comparisons between scores (B). CLIF-C OF = CLIF-consortium organ failure, CLIF-SOFA = chronic liver failure-SOFA, MELD = Model for End-Stage Liver Disease, qCLIF-SOFA = quick CLIF-SOFA, qSOFA = quick Sequential Organ Failure Assessment, SAPS II = Simplified Acute Physiology Score II, SIRS = systemic inflammatory response syndrome.

Predictive value of in-hospital mortality was significantly higher using CLIF-SOFA (AUROC, 0.742; 95% CI, 0.714–0.770), CLIF-C OF (AUROC, 0.741; 95% CI, 0.713–0.769), and SAPS II (AUROC, 0.759; 95% CI, 0.733–0.786) than SIRS criteria (AUROC, 0.618; 95% CI, 0.590–0.647), qSOFA (AUROC, 0.612; 95% CI, 0.584–0.640), MELD (AUROC, 0.632; 95% CI, 0.601–0.662), or qCLIF-SOFA (AUROC, 0.680; 95% CI, 0.650–0.710) (*P* < .05 for all) while CLIF-SOFA and CLIF-C OF were comparable (*P* = .871) (Fig. 2 and Table 5).

**Table 5:**
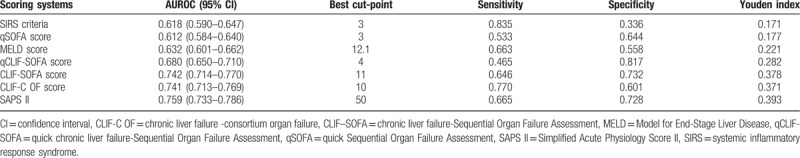
Prognostic accuracy of SIRS criteria, qSOFA, MELD, CLIF-SOFA, CLIF-C OF, qCLIF-SOFA, and SAPS II among critically ill cirrhotic patients with suspected infection.

**Figure 3:**
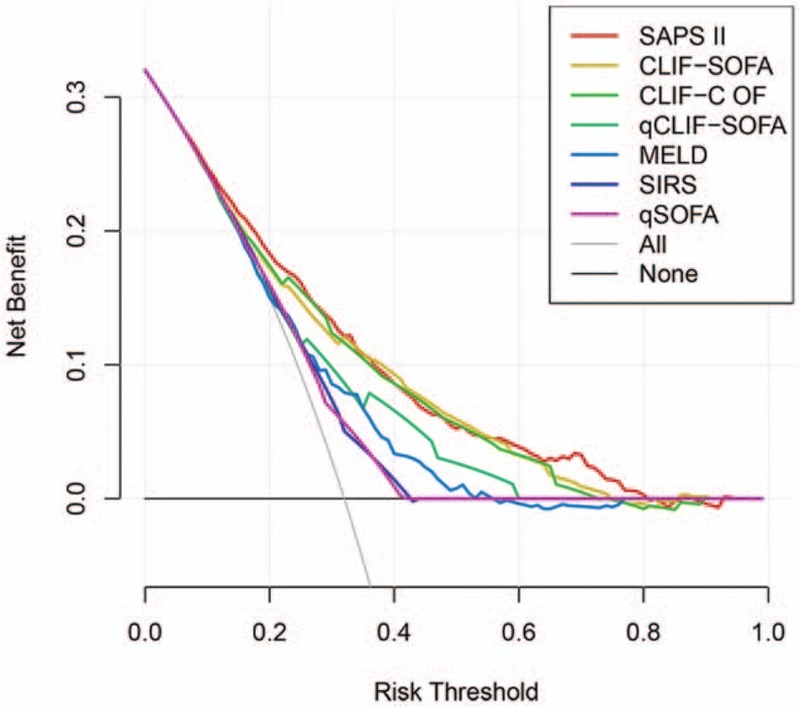
Decision curve depicting the benefit of each score based on the risk threshold. The gray curve depicts the net benefit of recommending the intervention to everyone in the cohort regardless of risk, while the black horizontal line indicates the net benefit (at net benefit of zero) of without intervention in the cohort. CLIF-C OF = CLIF-consortium organ failure, CLIF-SOFA = chronic liver failure-SOFA, MELD = Model for End-Stage Liver Disease, qCLIF-SOFA = quick CLIF-SOFA, qSOFA = quick Sequential Organ Failure Assessment, SAPS II = Simplified Acute Physiology Score II, SIRS = systemic inflammatory response syndrome.

To facilitate the comparison of clinical significance among different scoring systems, decision curve analysis was performed. In present analysis, CLIF-SOFA, CLIF-C OF, and SAPS II based models had higher net benefit than SIRS, qSOFA, MELD, or qCLIF-SOFA based models across a wide range of decision threshold probabilities (approximately 10%–70% risk of death) (Fig. 3 and Table 6). It seemed SIRS and qSOFA failed to produce net benefit while the probability of death exceeding more than 40%. However, at extremely high risks of death (> 70%), CLIF-SOFA and CLIF-C OF based models presented poor predictive value with negative net benefit (Fig. 3).

**Table 6:**
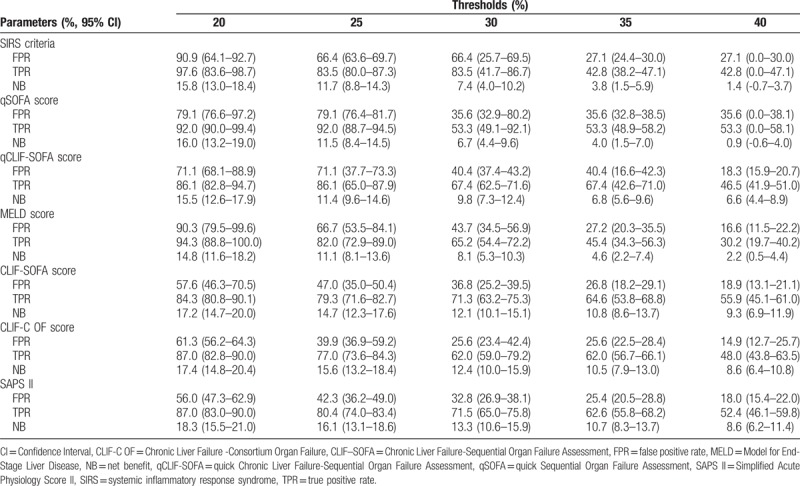
Net benefit, true- and false-positive rate of using SIRS, qSOFA, MELD, CLIF-SOFA, CLIF-C OF, qCLIF-SOFA, and SAPS II on different decision thresholds.

## 4 Discussion

Bacterial infections are responsible for the death of 30% to 50% cirrhotic patients.^[21]^ Thus, an optimal prognostic score helps ICU physicians to early identify those with high risk of death and to intervene timely. In this study, we compared the predictive value of five scoring systems on the prognosis of critically ill cirrhotic patients with suspected infection. We confirmed that CLIF-SOFA, CLIF-C OF, and SAPS II had superior prognostic value for in-hospital mortality than SIRS criteria, qSOFA, MELD, or qCLIF-SOFA. However, at extremely high risks of death, CLIF-SOFA and CLIF-C OF scores showed poor predictive ability.

Several scores had been developed to evaluate the severity of cirrhosis, mainly focusing on the loss of liver function and its complications. Previous studies proved that MELD score, had better accuracy in predicting 3-month mortality among patients with chronic liver disease and needing liver transplantation, compared with Child-Turcotte-Pugh (CTP) score, which was applied for the allocation of donor liver.^[16]^ However, a negative value of MELD scores may not be always favorable. Later, the Royal Free Hospital (RFH) score was proposed in an observational study including 312 cirrhotic patients in ICU and it proved similar discriminative ability compared with SOFA and CLIF-SOFA and better than MELD score.^[22]^ Another modified score for critically ill cirrhosis (MSCIC) including prothrombin time, bilirubin, vasopressin usage, HE and SIRS, was developed and demonstrated superior to CTP, MELD, Acute Physiology and Chronic Health Evaluation (APACHE) II scores, and CLIF-SOFA.^[23]^ However, the SIRS criteria within MSCIS were modified and it was also too complex to calculate.

The prognostic accuracy of liver-specific scoring systems were validated and favored by present study. CLIF-SOFA, a modified SOFA score, was customized for chronic hepatic disease including more hepatic components compared with SOFA. CLIF-C OF was a simplified version of CLIF-SOFA and presented similar performance with CLIF-SOFA in the previous study,^[24]^ which was consistent with our results. Bilirubin, HE, and INR, included in CLIF-SOFA and CLIF-C OF, were significantly associated with the prognosis of cirrhotic patients and were recommended to be used as predictor of outcomes.^[25]^ In this sense, CLIF-SOFA and CLIF-C OF were more appropriate for patients with chronic disease than SIRS or qSOFA. A large research had previously proved that SIRS was not a perfect score for predicting ICU mortality and had poor ability to define a transition point in risk of death.^[26]^ Some explained SIRS criteria were too sensitive and not specific enough that the severity of illness was overestimated.^[27]^ As our analysis indicated, SIRS showed nearly no prognostic value when risk probability surpassed 40%. Similar to SIRS criteria, qSOFA, with no laboratory measurements and originally developed to be used at bedside, also failed to predict and discriminate patients with high risk. Though showing great prognostic value among patients with suspected infection outside of the ICU,^[7,28]^ qSOFA may be inappropriate for those severe patients. Since lactate had long been regarded as an predictor for sepsis or septic shock, previous studies found that when adding lactate levels to qSOFA, the predictive value turned significantly elevated.^[29]^ To improve the predictive validity of qSOFA, some authors even recommended replacing mention status with lactate levels for mention alternation was potentially biased by physician's judgment and was thus difficult to validate.^[28]^ Nevertheless, this may result in concern that “quick” SOFA will become not that quick. Inspired by qSOFA, some authors argued that CLIF-SOFA was too time-consuming and proposed qCLIF-SOFA.^[12]^ Compared with CLIF-SOFA, HE grade was excluded and subscores of each component was just 0 or 1. Although they found simpler qCLIF-SOFA possessed comparable accuracy for prognostic prediction of 28- and 90-day mortality compared with CLIF-SOFA, the results were not verified in population out of ICU, such as emergency department. Moreover, risk factors and severe complication such as HE were not taken into account, which may result in missed diagnosis. It was worth mentioning that 2 of 5 components within qCLIF-SOFA were circulation-related (mean arterial pressure and vasopressin application) and it may cause some overlap and underestimate the illness of liver itself. However, as a classical severity score in ICU,^[17]^ SAPS II remained a better predictive value than MELD, which was consistent with a recent observational study.^[23]^

Although we supported the use of CLIF-SOFA and CLIF-C OF as screening tools for the in-hospital mortality of cirrhotic patients with suspected infection, they both showed poor prognostic value for patients with high risk of death (above mortality of 70% in present analysis showed in Fig. 3). This probably explained why the AUROC of CLIF-SOFA and CLIF-C OF were marginally smaller than those reported in previous studies.^[23,30,31]^ Jalan et al^[11]^ suggested adding age and log-transformed white blood cell count to CLIF-C OF to produce a specific prognostic score for acute-on-chronic liver failure (ACLF) named CLIF Consortium ACLF score (CLIF-C ACLFs). The CLIF-C ACLFs showed good discrimination ability at ACLF diagnosis and proved to have potential use on stratifying the risk of death in ACLF patients. Recently, authors discovered that CLIF-SOFA presented an improvement of discriminative ability when incorporate temperature into original CLIF-SOFA.^[32]^ It indicated that inflammatory factor had great impact weight on mortality and could complement the prognostic accuracy of CLIF-SOFA or CLIF-C OF.

There are several limitations for the present study. First, as a retrospective cohort research, the study may have a hereditary limitation. For example, we could not avoid the heterogeneity because the cohort included cirrhotic patients of various etiologies. Second, HE grade was roughly defined according Glasgow Coma Scale for the data on altered mention, nervous reflex was limited and some other items such as electroencephalogram were not available. Third, the study was based on a single-center database, which may result in concerns on the generalization of the conclusions and the selection bias.

In summary, although there are a lot of discussion and controversies on the predictive value of all kinds of prognostic scores, CLIF-SOFA, CLIF-C OF, and SAPS II scoring systems are optimal tools to predict the prognosis of critically ill cirrhotic patients with suspected infection up to now. However, large multicenter prospective studies are needed to improve the predictive ability of scoring systems.

## Author contributions

PL and J-CZ designed the study, extracted, and analyzed the data and drafted the article. PL, S-JW, Q-CS, YF, TC, Y-XY, K-HP, LL, J-CZ, and Y-SY participated in the study and reviewed the article for critical content.

**Conceptualization:** Jian-Cang Zhou.

**Data curation:** Ying Fu.

**Formal analysis:** Peng Lan.

**Investigation:** Qing-Ye Xu.

**Methodology:** Peng Lan, Yun-Xian Yu.

**Project administration:** Jian-Cang Zhou.

**Resources:** Shuo-Jia Wang, Qiu-Cheng Shi.

**Software:** Peng Lan.

**Supervision:** Jian-Cang Zhou.

**Validation:** Tao Chen, Ling Lin.

**Visualization:** Peng Lan.

**Writing – original draft:** Peng Lan, Kong-Han Pan.

**Writing – review & editing:** Jian-Cang Zhou, Yun-Song Yu.

## References

[1] Gustot T Fernandez J Szabo G Sepsis in alcohol-related liver disease. J Hepatol 2017;67:1031–50. 28647569 10.1016/j.jhep.2017.06.013

[2] Fernandez J Acevedo J Castro M Prevalence and risk factors of infections by multiresistant bacteria in cirrhosis: a prospective study. Hepatology 2012;55:1551–61. 22183941 10.1002/hep.25532

[3] Noor MT Manoria P Immune dysfunction in cirrhosis. J Clin Transl Hepatol 2017;5:50–8. 28507927 10.14218/JCTH.2016.00056 PMC5411357

[4] Winters BD Eberlein M Leung J Long-term mortality and quality of life in sepsis: a systematic review. Crit Care Med 2010;38:1276–83. 20308885 10.1097/CCM.0b013e3181d8cc1d

[5] Singer M Deutschman CS Seymour CW The Third International Consensus Definitions for Sepsis and Septic Shock (Sepsis-3). JAMA 2016;315:801–10. 26903338 10.1001/jama.2016.0287 PMC4968574

[6] Raith EP Udy AA Bailey M Prognostic accuracy of the SOFA score, SIRS criteria, and qSOFA score for in-hospital mortality among adults with suspected infection admitted to the intensive care unit. JAMA 2017;317:290–300. 28114553 10.1001/jama.2016.20328

[7] Freund Y Lemachatti N Krastinova E Prognostic accuracy of sepsis-3 criteria for in-hospital mortality among patients with suspected infection presenting to the emergency department. JAMA 2017;317:301–8. 28114554 10.1001/jama.2016.20329

[8] Cheng B Li Z Wang J Comparison of the performance between sepsis-1 and sepsis-3 in ICUs in China: a retrospective multicenter study. Shock 2017;48:301–6. 28448400 10.1097/SHK.0000000000000868 PMC5516667

[9] Malinchoc M Kamath PS Gordon FD A model to predict poor survival in patients undergoing transjugular intrahepatic portosystemic shunts. Hepatology 2000;31:864–71. 10733541 10.1053/he.2000.5852

[10] Moreau R Jalan R Gines P Acute-on-chronic liver failure is a distinct syndrome that develops in patients with acute decompensation of cirrhosis. Gastroenterology 2013;144:1426–37. 1437 e1-9. 23474284 10.1053/j.gastro.2013.02.042

[11] Jalan R Saliba F Pavesi M Development and validation of a prognostic score to predict mortality in patients with acute-on-chronic liver failure. J Hepatol 2014;61:1038–47. 24950482 10.1016/j.jhep.2014.06.012

[12] Zhou XD Zhang JY Liu WY Quick chronic liver failure-sequential organ failure assessment: an easy-to-use scoring model for predicting mortality risk in critically ill cirrhosis patients. Eur J Gastroenterol Hepatol 2017;29:698–705. 28240612 10.1097/MEG.0000000000000856

[13] Zhang Z Ji X Quadratic function between arterial partial oxygen pressure and mortality risk in sepsis patients: an interaction with simplified acute physiology score. Sci Rep 2016;6:35133. 27734905 10.1038/srep35133 PMC5062070

[14] Hassanein T Blei AT Perry W Performance of the hepatic encephalopathy scoring algorithm in a clinical trial of patients with cirrhosis and severe hepatic encephalopathy. Am J Gastroenterol 2009;104:1392–400. 19455117 10.1038/ajg.2009.160

[15] Liotta EM Lizza BD Romanova AL 23.4% Saline decreases brain tissue volume in severe hepatic encephalopathy as assessed by a quantitative CT marker. Crit Care Med 2016;44:171–9. 26308431 10.1097/CCM.0000000000001276 PMC5243943

[16] Le Gall JR Lemeshow S Saulnier F A new Simplified Acute Physiology Score (SAPS II) based on a European/North American multicenter study. JAMA 1993;270:2957–63. 8254858 10.1001/jama.270.24.2957

[17] Wiesner R Edwards E Freeman R Model for end-stage liver disease (MELD) and allocation of donor livers. Gastroenterology 2003;124:91–6. 12512033 10.1053/gast.2003.50016

[18] Hijazi Z Oldgren J Lindbäck J The novel biomarker-based ABC (age, biomarkers, clinical history)-bleeding risk score for patients with atrial fibrillation: a derivation and validation study. Lancet 2016;387:2302–11. 27056738 10.1016/S0140-6736(16)00741-8

[19] Den RB Yousefi K Trabulsi EJ Genomic classifier identifies men with adverse pathology after radical prostatectomy who benefit from adjuvant radiation therapy. J Clin Oncol 2015;33:944–51. 25667284 10.1200/JCO.2014.59.0026 PMC4884273

[20] Kerr KF Brown MD Zhu K Assessing the clinical impact of risk prediction models with decision curves: guidance for correct interpretation and appropriate use. J Clin Oncol 2016;34:2534–40. 27247223 10.1200/JCO.2015.65.5654 PMC4962736

[21] Tandon P Garcia-Tsao G Bacterial infections, sepsis, and multiorgan failure in cirrhosis. Semin Liver Dis 2008;28:26–42. 18293275 10.1055/s-2008-1040319

[22] Theocharidou E Pieri G Mohammad AO The Royal Free Hospital score: a calibrated prognostic model for patients with cirrhosis admitted to intensive care unit. Comparison with current models and CLIF-SOFA score. Am J Gastroenterol 2014;109:554–62. 24492755 10.1038/ajg.2013.466 PMC3978197

[23] Bao Q Wang B Yu L A modified prognostic score for critically ill patients with cirrhosis: an observational study. J Gastroenterol Hepatol 2016;31:450–8. 26251873 10.1111/jgh.13076

[24] Arroyo V Moreau R Jalan R Acute-on-chronic liver failure: a new syndrome that will re-classify cirrhosis. J Hepatol 2015;62:S131–143. 25920082 10.1016/j.jhep.2014.11.045

[25] D’Amico G Garcia-Tsao G Pagliaro L Natural history and prognostic indicators of survival in cirrhosis: a systematic review of 118 studies. J Hepatol 2006;44:217–31. 16298014 10.1016/j.jhep.2005.10.013

[26] Kaukonen KM Bailey M Pilcher D Systemic inflammatory response syndrome criteria in defining severe sepsis. N Engl J Med 2015;372:1629–38. 25776936 10.1056/NEJMoa1415236

[27] Vincent JL Martin GS Levy MM. qSOFA does not replace SIRS in the definition of sepsis. Crit Care 2016;20:210. 27423462 10.1186/s13054-016-1389-z PMC4947518

[28] Seymour CW Liu VX Iwashyna TJ Assessment of clinical criteria for sepsis: for the Third International Consensus Definitions for Sepsis and Septic Shock (Sepsis-3). JAMA 2016;315:762–74. 26903335 10.1001/jama.2016.0288 PMC5433435

[29] Escobar GJ Gardner MN Greene JD Risk-adjusting hospital mortality using a comprehensive electronic record in an integrated health care delivery system. Med Care 2013;51:446–53. 23579354 10.1097/MLR.0b013e3182881c8e

[30] Sy E Ronco JJ Searle R Prognostication of critically ill patients with acute-on-chronic liver failure using the Chronic Liver Failure-Sequential Organ Failure Assessment: a Canadian retrospective study. J Crit Care 2016;36:234–9. 27569253 10.1016/j.jcrc.2016.08.003

[31] Li N Huang C Yu K-K Validation of prognostic scores to predict short-term mortality in patients with HBV-related acute-on-chronic liver failure. Medicine 2017;96:e6802. 28445322 10.1097/MD.0000000000006802 PMC5413287

[32] Zhou XD Chen QF Wang ZX Acute circulatory failure-chronic liver failure-sequential organ failure assessment score: a novel scoring model for mortality risk prediction in critically ill cirrhotic patients with acute circulatory failure. Eur J Gastroenterol Hepatol 2017;29:464–71. 28030513 10.1097/MEG.0000000000000817

